# A case of concurrent occurrence of carcinoma showing thymus-like differentiation and follicular variant of papillary thyroid cancer in the same thyroid

**DOI:** 10.1093/jscr/rjab570

**Published:** 2022-01-15

**Authors:** Takahito Kimura, Keisuke Enomoto, Masamitsu Kono, Masanobu Hiraoka, Saori Takeda, Naoko Kumashiro, Shun Hirayama, Eri Kimura, Shunji Tamagawa, Makiko Ohtani, Shin-Ichi Murata, Muneki Hotomi

**Affiliations:** Department of Otorhinolaryngology-Head and Neck Surgery, Wakayama Medical University, 811-1 Kimiidera, Wakayama 641-8509, Japan; Department of Otorhinolaryngology-Head and Neck Surgery, Wakayama Medical University, 811-1 Kimiidera, Wakayama 641-8509, Japan; Department of Otorhinolaryngology-Head and Neck Surgery, Wakayama Medical University, 811-1 Kimiidera, Wakayama 641-8509, Japan; Department of Otorhinolaryngology-Head and Neck Surgery, Wakayama Medical University, 811-1 Kimiidera, Wakayama 641-8509, Japan; Department of Otorhinolaryngology-Head and Neck Surgery, Wakayama Medical University, 811-1 Kimiidera, Wakayama 641-8509, Japan; Department of Otorhinolaryngology-Head and Neck Surgery, Wakayama Medical University, 811-1 Kimiidera, Wakayama 641-8509, Japan; Department of Otorhinolaryngology-Head and Neck Surgery, Wakayama Medical University, 811-1 Kimiidera, Wakayama 641-8509, Japan; Department of Otorhinolaryngology-Head and Neck Surgery, Wakayama Medical University, 811-1 Kimiidera, Wakayama 641-8509, Japan; Department of Otorhinolaryngology-Head and Neck Surgery, Wakayama Medical University, 811-1 Kimiidera, Wakayama 641-8509, Japan; Department of Otorhinolaryngology-Head and Neck Surgery, Wakayama Medical University, 811-1 Kimiidera, Wakayama 641-8509, Japan; Department of Diagnostic Pathology, Wakayama Medical University, 811-1 Kimiidera, Wakayama 641-8509, Japan; Department of Otorhinolaryngology-Head and Neck Surgery, Wakayama Medical University, 811-1 Kimiidera, Wakayama 641-8509, Japan

## Abstract

Carcinoma showing thymus-like differentiation (CASTLE) is a rare thyroid cancer. This is the first report of a case of concurrent occurrence of CASTLE with papillary thyroid carcinoma (PTC).

A 66-year-old male patient had hoarseness with right vocal cord paralysis. Ultrasonography revealed a hypoechoic nodule in the inferior pole of the right thyroid lobe. Ultrasound-guided fine-needle aspiration cytology suggested differentiated thyroid cancer. The patient underwent total thyroidectomy with neck dissection. Pathological examination revealed two different thyroid cancers: a CASTLE and a follicular variant of PTC. Postoperative radiation therapy was performed. The patient was still alive after 5 year following the initial treatment without evidence of recurrence. The oncological management of patients with concurrent occurrence of different thyroid cancers should consider the biological behavior of both tumors.

## INTRODUCTION

Carcinoma showing thymus-like differentiation (CASTLE) is a very rare form of thyroid cancer [1–6]. The tumor has been clinicopathologically classified in the WHO classification of thyroid tumors, fourth edition [1]. CASTLE resembles thymic epithelial tumors, with a predilection for the inferior pole of the thyroid and it is thought to be derived from ectopic thymic tissue or thymic remnant tissue during the fetal period, and it is embryologically different from papillary thyroid cancer, which defined as a carcinoma derived from thyroid follicular cells. In this case report, we present a rare case of concurrent occurrence of CASTLE and papillary thyroid carcinoma (PTC).

## CASE

**Figure 1:**
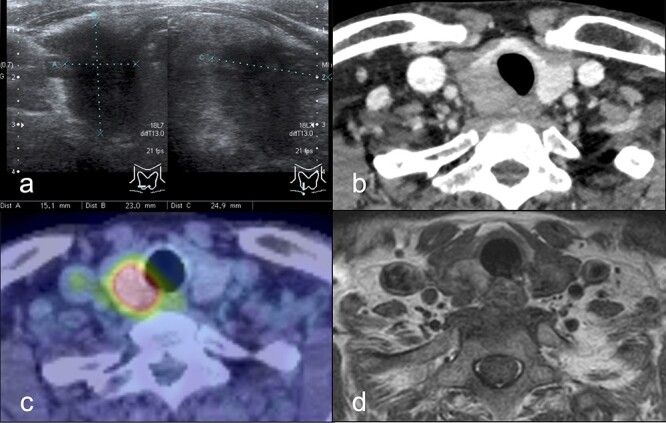
(**a**) Cervical ultrasound examination: a hypoechoic nodule with indistinct borders and irregular margins (15 × 23 × 25 mm) was found at the inferior pole of the right lobe. (**b**) Dynamic CT scan: a mass lesion (18 × 24 × 26 mm) was found in the inferior pole of the right lobe thyroid gland without contrast effect in equilibrium phase. (**c**) PET/CT scan: FDG hyperaccumulation was observed in a nodule (~25 mm diameter) at the inferior pole of the right lobe of the thyroid gland. (**d**) MRI scan: A mass (30 mm diameter) with mildly high signal on Dixon-T2-weighted imaging was found at the inferior pole of the right lobe of the thyroid gland, and tracheal invasion. Extracapsular invasion was suspected. Abbreviations: CT, computed tomography; PET, positive emission tomography; FDG, fluorodeoxyglucose; MRI, magnetic resonance imaging.

A 66-year-old man with hoarseness and coughing for more than a month was referred to our hospital for further investigation. He also had a palpable mass at the right thyroid lobe. Blood examinations showed no abnormalities in thyroid function (Table 1). Ultrasonography showed a hypoechoic nodule (15 × 23 × 25 mm) at the inferior pole of the right thyroid lobe (Fig. 1a) with bilateral enlarged cervical lymph nodes (10 mm). Contrast-enhanced computed tomography (CT) revealed the mass (18 × 24 × 26 mm) in equilibrium phase without contrast effect (Fig. 1b). The 2-deoxy-2-[^18^F] fluoro-D-glucose positron emission tomography showed high accumulation in the mass, whereas the cervical lymph nodes did not show accumulations (Fig. 1c). Magnetic resonance imaging showed the 30 mm diameter mass with mildly high signal on Dixon-T2-weighted imaging and suggested tracheal invasion and extraparenchymal extension (Fig. 1d). Flexible laryngeal fiberscopy showed right vocal cord paralysis. Ultrasound-guided fine-needle aspiration cytology indicated an atypical cell mass with a higher cell density and cumulation from the thyroid tumor, which was dominantly composed of small follicular structures without apparent papillary structures. However, the cells showed nuclear groove-like irregularities and intranuclear vacuoles, suggesting differentiated thyroid cancer.

**Table 1:** Blood examination

WBC	5950/μl	CRP	0.12 mg/dl
Hb	15.7 g/dl	Na	137 mEq/L
PLT	21.8 × 10^4^/μl	K	4.5 mEq/L
Total protein	7.2 g/dl	Cl	101 mEq/L
Albumin	4.7 g/dl	TSH	1.004 μIU/ml
AST(GOT)	22 IU/L	FT4	1.11 ng/dl
ALT(GPT)	22 IU/L	FT3	3.03 ng/dl
LD	180 IU/L	Tg	17.9 ng/ml
Creatinine	0.86 mg/dl	Tg-Ab	<10.0 IU/ml
Glucose	110 mg/dl	TPO-Ab	5.4 IU/ml

The patient was finally diagnosed as differentiated thyroid cancer (cT4aN0M0, right recurrent laryngeal nerve and extrathyroidal invasion). A total thyroidectomy with neck dissection of the central area and right lateral cervical lymph nodes was performed. The thyroid cancer invaded the right recurrent laryngeal nerve, the membranous trachea and the esophageal envelope.

**Figure 2:**
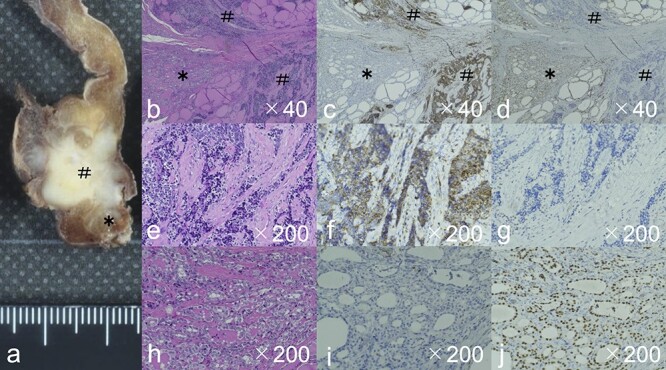
The gross image (**a**) showed a white mass with very close Carcinoma showing thymus-like differentiation, CASTLE (#) and papillary thyroid carcinoma, PTC (*). HE staining showed the CASTLE had chordate and small enriched structures (**b**), and the tumor cells showed a very high N/C ratio, large oval nuclei, chromatin densification, and often lymphocytic infiltration around the tumor cells (**e**). Immunostaining also showed CD5 positivity in CASTLE (**c**, **f**), but negativity in PTC (**c**, **i**). PTC showed small to medium-sized follicular structures by HE staining (**b**). Tumor cells showed intranuclear inclusion body, a nuclear groove resembling a coffee bean, and ground-glass appearance (**h**). Immunostaining showed TTF-1 positivity in PTC (**d**, **j**), but negativity in CASTLE (**d**, **g**). Abbreviations: CASTLE, Carcinoma showing thymus-like differentiation; PTC: papillary thyroid carcinoma; HE stain, hematoxylin and eosin stain; TTF-1, thyroid transcription factor-1.

Gross examination of the excised specimen exhibited two tumors in a white mass at the inferior pole of the right thyroid lobe (Fig. 2). Pathological examination with Hematoxylin and Eosin (HE) staining showed that the large tumor located cephalad had chordated and small enriched structures (Fig. 2b). The first tumor was diagnosed as CASTLE according to the results of immunostaining evaluation: CD5 positive, p63 positive, CK5/6 positive, Ki-67 labeling index (20%–30%, hot spot), TTF-1 negative, calcitonin negative and thyroglobulin negative (Fig. 2c, d, f, g). The other tumor, which was located caudally, was shown to be a small to medium-sized follicular structure with a body of intranuclear inclusion, a nuclear groove resembling a coffee bean, and ground-glass appearance (Fig. 2b, h). The tumor was diagnosed as follicular variant of PTC based on the Immunostaining finding of CD5-negative and TTF-1-positive (Fig. 2c, d, i, j). The cervical lymph nodes showed metastatic findings of CASTLE in the right paratracheal region and in the upper–inner deep cervical region.

The postoperative staging of CASTLE was pT4a (recurrent laryngeal nerve invasion, tracheal invasion and esophageal invasion) pN1b, and PTC was pT1a, pN0. The PTC was localized within the thyroid gland without metastasis. External beam radiation therapy with 60Gy was done for the CASTLE as an additional postoperative treatment. The patient was still alive after 5 years of follow-up without evidence of recurrence.

## DISCUSSION

CASTLE was first proposed by in 1985 as intrathyroidal epithelial thymoma (ITET) [7]. Then, in 1991, it was named CASTLE [8] and registered in the WHO classification in 2004 [9]. The incidence of CASTLE is very low, ranging between 0.1% and 0.15% [10]. Most cases are published as case reports [2–6].

CASTLE typically occurs at the inferior pole of the thyroid gland. Histopathological-specific features of CASTLE are tumor cells with an island-like structure, stroma composed of dense fibrous connective tissue, infiltration of lymphocytes and plasma cells, polygonal or spindle-shaped cells with large prominent nuclei with indistinct cell borders, a tendency of squamous epithelium differentiation, and expression of CD5 [11]. CD5 is a well-known marker for differential diagnosis of CASTLE because thymoma, anaplastic thyroid carcinoma and PTC derived from follicular cells do not express the marker. Ito *et al.* reported that 82% of patients with CASTLE were positive for CD5 [12]. In the current case, we could diagnose the tumor in the inferior pole of the thyroid as CASTLE based on the findings of squamous cell-like differentiation, lymphocytic infiltration around the tumor cells, and expression of CD5. A follicular variant of PTC was incidentally diagnosed by pathological examination that indicated a small to medium-sized follicular structure with intranuclear inclusion body, a nuclear groove resembling a coffee bean, and ground-glass appearance, and expression of TTF-1.

This is the first case reporting a coexistence of CASTLE and PTC in the same thyroid. There was no pathological correlation between CASTLE and PTC in this case. Also, there was no characteristic family history or genetic component. Thus, we concluded that this case was an incidental finding of the coexistence of two different types of thyroid cancers. There have been only two cases of CASTLE that reported to have an incidental carcinoma [12].

Total thyroidectomy with neck dissection is the gold standard for the treatment of CASTLE [12, 13]. External radiation after surgery contributes to reduce local recurrence [14, 15]. Meanwhile, total thyroidectomy with/without postoperative radioactive iodine is the standard therapeutic strategy for PTC. In the current case, the PTC was diagnosed as pT1a without cervical lymph node metastases. We performed external radiation following total thyroidectomy with neck dissection in this patient. As results, there has been no recurrence for 5 years. There are currently no definite treatment guidelines for patients with double thyroid cancer of CASTLE and PTC. Therapeutic strategy should consider tumor progression.

## CONCLUSION

Our patient was successfully treated for double thyroid cancer of CASTLE and PTC. Therapeutic strategy should consider tumor progression of both CASTLE and PTC.
